# 2548. Pharmacokinetic Analysis of Co-Administering Doravirine, Tenofovir Disoproxil Fumarate, Estradiol, and Spironolactone

**DOI:** 10.1093/ofid/ofad500.2165

**Published:** 2023-11-27

**Authors:** Kevin Lam, Tingting Zhan, Walter Kraft, Edwin Lam

**Affiliations:** Thomas Jefferson University, Philadelphia, Pennsylvania; Thomas Jefferson University, Philadelphia, Pennsylvania; Thomas Jefferson University, Philadelphia, Pennsylvania; Janssen Research & Development LLC, Spring House, Pennsylvania

## Abstract

**Background:**

Despite the availability of doravirine (DOR) as a fixed-dose combination product and its favorable safety profile, transgender women (TGW) are less likely to adhere to antiretroviral therapy over concerns of drug interactions with feminizing hormone therapy. This study evaluated the effect of DOR (given as a single dose containing lamivudine (3TC) and tenofovir disoproxil fumarate (TDF)) and TDF on the pharmacokinetics (PK) of estradiol (E2) and spironolactone and vice versa.

**Methods:**

A phase I, three-period crossover, bi-directional drug interaction study was conducted. Healthy volunteers self-identified as TGW were randomized 1:1 into 2 sequences containing treatment A (DOR/3TC/TDF 100 mg/300 mg/300 mg as a single dose), treatment B (E2 4 mg twice daily, spironolactone 200 mg twice daily, and placebo for one day), and treatment C (single dose of DOR/3TC/TDF 100 mg/300 mg/300 mg, E2 4 mg twice daily, and spironolactone 200 mg twice daily for one day). Plasma DOR, TDF, E2, and testosterone were collected for PK evaluation. DOR, TDF, and E2 PK parameters were computed via a non-compartmental analysis and compared using a paired student’s t-test. Safety and tolerability were evaluated throughout the study.

Study Flow

**Figure:**
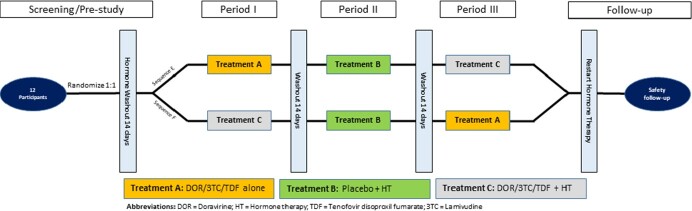

**Results:**

Out of 8 participants enrolled in the study, 6 completed the study. Geometric mean ratios (co-administration vs. without co-administration) and 90% confidence intervals for DOR, TDF, and E2 area under the concentration-time curve from zero to last measured concentration (AUC_0-last_), maximum concentration (C_max_), and concentration at 24 hours (C_24_) are reported in Table 1. Log-transformed DOR, TDF, and E2 PK parameters computed with and without co-administration were not statistically significant (p > 0.05).

Statistical Analysis of PK Parameters for Doravirine, Tenofovir Disoproxil Fumarate, and Estradiol

**Figure:**
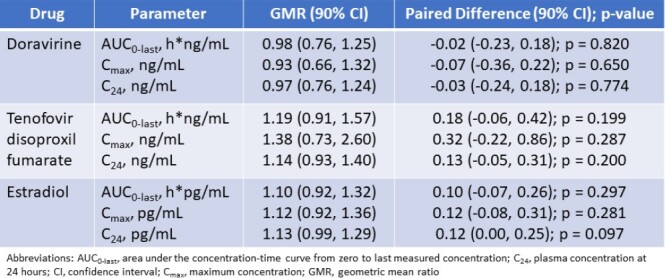

**Conclusion:**

These results demonstrate that a clinically significant interaction is not observed involving the use of feminizing hormones on DOR/TDF PK. Similarly, no observed impact on estradiol PK and testosterone is anticipated following use of DOR/TDF/3TC. DOR/TDF administered with and without E2 and spironolactone was generally well tolerated.

This work was supported by an Investigator Studies Program grant provided by Merck & Co., Inc. MISP59198. The opinions expressed are those of the authors and do not necessarily represent those of Merck & Co., Inc.

**Disclosures:**

**All Authors**: No reported disclosures

